# Development and Evaluation of Dual Cross-Linked Pulsatile Beads for Chronotherapy of Rheumatoid Arthritis

**DOI:** 10.1155/2013/906178

**Published:** 2012-12-09

**Authors:** Abanesh kumar Bansal, Vishal Pande

**Affiliations:** Department of Pharmaceutics, H.R. Patel Institute of Pharmaceutical Education and Research, Dhule, Shirpur 425405, Maharashtra, India

## Abstract

In the present investigation, pulsatile release beads were prepared by ionic gelation technique. Lornoxicam dual cross-linked beads were prepared by dropping dispersed phase of lornoxicam, pectin, and sodium alginate into the dispersion phase of different concentrations of calcium chloride solution followed by aluminium chloride solution. The formulated beads were further coated by Eudragit L & S 100 in the ratio 1 : 2 w/w in order to achieve desired lag time. *In vitro* release study showed lag time of 5–8 h before release of lornoxicam from the formulated beads. Thus, formulated dual cross-linked beads when administered at bed time may release lornoxicam when needed most for chronotherapeutics of early morning rheumatoid arthritis attacks in chronic patients.

## 1. Introduction

Lornoxicam is the nonsteroidal anti-inflammatory drug with potent anti-inflammatory and analgesic property [1]. Lornoxicam has greater potency than the other oxicam derivatives such as tenoxicam and piroxicam as cyclooxygenase (COX)-1 inhibitors [2, 3]. Many diseases such as cardiovascular disease, bronchial asthma, rheumatoid arthritis, peptic ulcer, diabetes mellitus, and sleep disorders show circadian rhythms in their pathophysiology with prevailing night or early-morning manifestations. These diseases demand pulsatile drug delivery approach where the drug releases after depicting lag phase [4–6]. In this research work dual cross-linked pulsatile beads of lornoxicam were prepared by ionotropic gelation method using calcium chloride, aluminum chloride, pectin, and sodium alginate [7].

Alginate is a naturally occurring linear biopolymer which is found in brown seaweeds such as *Laminaria hyperborean, Ascophyllum nodosum, *and* Macrocystis pyrifera*. Alginate is found as mixed salt of various cations such as Al^3+^, Mg^2+^, Sr^2+^, Ba^2+^, and Na^+^ [8]. Alginic acid containing 1,4′-linked D-mannuronic acid and L-guluronic acid units was arranged in block as the same unit or random sequence of these units [9]. These arrangements form egg-box-like structure on contacting with divalent calcium ion and form hydrogel bead in aqueous solution of calcium chloride [10]. L-guluronic acid units of alginate bind with the divalent cation in solution and form gel structure. In this bead-like structure various biomolecules such as protein, peptide, cell, and drug can be entrapped. Sodium alginate controls or sustains the release of drug from gel matrix of the beads. The release of payload from alginate matrix can be achieved either by diffusion or alginate degradation or both [8].

Pectin is heterogeneous polysaccharides which are found in the cell wall of the plants. Pectin containing linear chain of poly(D-galacturonic acid) is bonded via 1,4-glycosidic linkage [11]. Solubility of pectin depends on the degree of methoxylation. As the degree of methoxylation increases, solubility of pectin decreases. Low methoxylated pectin has more capability to form insoluble rigid gel with divalent cation such as calcium ions cross-linked with galacturonic acid chain [12]. Pectin has pharmaceutical importance such as gelling or thickening agent and excipient for pharmaceutical purposes [13]. Hydration of pectin is deducted due to the increase of gelling property in the presence of acid, so incorporation of water reduces into the rigid gel structure. Pectin is not digested in stomach by enzyme such as proteases and amylase but it is sensitive to the enzyme which is secreted by microflora in the colon [14]. In this research work we reveal the application of dual cross-linked pulsatile beads to control the drug delivery. The disorders such as cardiovascular disease, bronchial asthma, rheumatoid arthritis, peptic ulcer, and so forth demand the pulsatile delivery of therapeutic agent. Natural polymers such as sodium alginate and pectin were used to formulate the dual cross-linked pulsatile beads by ionotropic gelation method using lornoxicam as a model drug. These polymers swell at higher pH and release the drug by diffusion or degradation mechanism in sustained release or burst release after predetermined lag time. These beads fill in the capsule and they are coated with Eudragit S100 & L100 as reported in literature. For achieving the required lag time, capsule was coated with Eudragit S100 & Eudragit L100 in proportion of 1 : 2 ratio which have the maximum solubility at 6.8 pH. Therefore, this combination of two polymers was selected for coating of dual cross-linked beads.

## 2. Materials and Methods 

### 2.1. Material

Lornoxicam was obtained as a gift sample from Glenmark Pharmaceutical Ltd. (Nashik, India). Sodium alginate and pectin (LOBA Chemie, Mumbai, India), Eudragit S100 and Eudragit L100 (Research Lab, Fine chemicals, Mumbai, India), Calcium chloride and Aluminum chloride anhydrous (Merck specialties private limited, Mumbai, India). All other were reagents used in this study were of analytical grade.

### 2.2. Solubility of Lornoxicam in Different pH Conditions

The solubility study was conducted to depict the solubility of lornoxicam at varying pH conditions. Saturated solutions of lornoxicam were prepared by dispersing an excess amount of drug in the buffer with pH 1.2, 6.8, 7.4, 12, and 13. The samples were subjected to orbital shaker (Remi Instrument Ltd. CIS-24) for 72 hours to aid maximum dissolution of the drug. After the incubation period, clear saturated solutions were obtained by filtration (0.45 *μ*m Millipore filter paper) and the concentration of the drug was determined spectrophotometrically (UV-1800 PC Shimadzu, Japan) at the corresponding wavelengths, after the appropriate dilution in the corresponding pH buffer.

### 2.3. Preparation of Single Cross-Linked Ca^++^ Pectin-Alginates Beads

Lornoxicam was dispersed in distilled water and sonicated for 45 sec for reducing the particle side of drug (Table 1). Preweighted amount of pectin and sodium alginate were completely dissolved into the above solution and stirred for few minutes. The uniform dispersion was carried out by dispersion dropping with 25 gauge needles into 5% calcium chloride solution and stirred for 10 min for cross-linking of pectin and alginate. After the required cross-linking time, beads were filtered, washed with 50 mL double distilled water, and dried in the incubator at 40°C till the weight is constant.

### 2.4. Preparation of Dual Cross-Linked Ca^2+^/Al^3+^ Pectin-Alginates Beads

Prepared Ca^2+^ single cross-linked pectin alginate beads (P6 and P7) were immediately transferred into a 50 mL solution of 5% aluminum chloride (% w/v) for 5 min and then the Ca^2+^ and Al^3+^ dual cross-linked beads were separated by filtration, washed thrice with 50 mL double distilled water, air dried, and finally vacuum-dried at 40°C till constant weight.

### 2.5. Preparation of Enteric-Coated Capsule

Enteric-coated gelatin capsules were prepared by manual dipping technique. Entire hard gelatin capsule was dipped in 2% w/v Eudragit S100 & Eudragit L100 (1 : 2 ratio) in methanol. The coated gelatin capsule was dried at 40°C for 30 min. Equivalent amount (8 mg) of dual cross-linked beads were filled along with 10% magnesium stearate as a lubricant in size 2 of enteric-coated capsule.

## 3. *In Vitro* Characterization of Formulated Pectin-Alginate Beads

### 3.1. Particle Size Analysis

Particle size analysis of dual cross-linked pectin alginate was performed using motic microscopy. Formulated 20 beads of each batches were randomly selected and observed in motic DMWB2-223 digital microscope fitted with 1/3 CCD camera imaging accessory and using Motic Images 2000 (1.3 Version) image analysis software.

### 3.2. Determination of Yield

The production yield of prepared dual cross-linked pectin-alginate beads was calculated using the weight of final product after drying with respect to the initial total weight of the drug and polymer used for preparation of beads and percent yields were calculated as per the following formula:

(1) $$\begin{array}{c} \%\text{Yield}\;=\frac{Pm}{Tm}\times 100, \end{array}$$

where *Pm* and *Tm* are practical yield and theoretical yield of formulation, respectively.

### 3.3. Drug Content

For determining drug content, weighed amount (35 mg) of dual cross-linked pectin-alginate beads were placed into the 100 mL of 0.1 N NaoH solution of each formulation and stirred the solution with mechanical stirrer then filtered the solution and analyzed spectrophotometrically at 377.5 nm in UV spectrophotometer [15]. % Entrapment efficiency was estimated by

(2) $$\begin{array}{c} \text{Entrapment Efficiency}\;\left(\%\right)\;=\frac{Aq}{Tq}\times 100, \end{array}$$

where *Aq* and *Tq* are actual quantity and theoretical quantity of present in formulation, respectively.

### 3.4. Swelling Study

Swelling behavior of pectin alginate dual cross-linked beads was studied by using digital caliper [16, 17]. All formulated batches were placed in 1.2 pH buffer and 6.8 pH buffer in Petridis and the diameter of 10 beads was measured after and before the swelling of beads at predetermined time interval, and swelling (%) was estimated according to the following relationship:

(3) $$\begin{array}{c} \text{Swelling}\;\left(\%\right)\;=\frac{D_{t}-D_{o}}{D_{o}}\times \;100, \end{array}$$

where *D*_*o*_ and *D*_*t*_ are the initial diameter of the beads and the diameter of the beads at a given time, respectively.

### 3.5. Scanning Electron Microscopy (SEM)

Morphological analysis or surface topography of dual cross-linked pectin alginate beads was carried out using scanning electron microscope. Beads were coated with thin palladium layer and surface topography was analyzed at 10 KV acceleration voltages using scanning electron microscope (JEOL JSM-6390, Tokyo, Japan).

### 3.6. Fourier Transforms Infrared (FTIR) Study

FTIR spectra of lornoxicam, physical mixture, and final formulation were recorded with FTIR spectrophotometer (IR IFFINITY-1 CE, Shimadzu corps, Japan) equipped with pyroelectric detector using dispersion method. The FTIR measurements were performed in the scanning range of 4,000–400 cm^−1^ at ambient temperature. The spectra were saved using IR solution software. All samples were softly grounded before analysis.

### 3.7. Differential Scanning Colorimetry (DSC)

DSC thermograms of lornoxicam, physical mixture, and pectin alginate beads were recorded using differential scanning colorimeter (DSC-60, Shimadzu, Japan). Each sample (5–10 mg) was scanned in pierced Al pans. The measurement was performed between 50 and 400°C at heating rate 10°C/min.

### 3.8. *In Vitro* Dissolution Studies

USP dissolution apparatus I (Dissolution test TDT-08L plus, Electrolab, India) was used to perform the release of dual cross-linked beads. Basket was rotated at 50 rpm and temperature maintained at  37.0 ± 0.5°C. Dissolution studies were carried out in 900 mL of 0.1 N hydrochloric acid buffer, 1.2 pH for 2 hour and pH 6.8 phosphate buffer for remaining hours. After 2 hours 1.2 pH hydrochloric acid buffer was replaced with 6.8 pH phosphate buffer and study was carried out for 12 hours. Samples (5 mL) were withdrawn and filtered. The withdrawal sample was replaced with equal volume of fresh medium at regular time interval. The amount of drug release was analyzed spectrophotometrically at 378 nm.

## 4. Results and Discussion

### 4.1. Solubility of Lornoxicam at Different pH Ranges at Room Temperature

**Figure 1:**
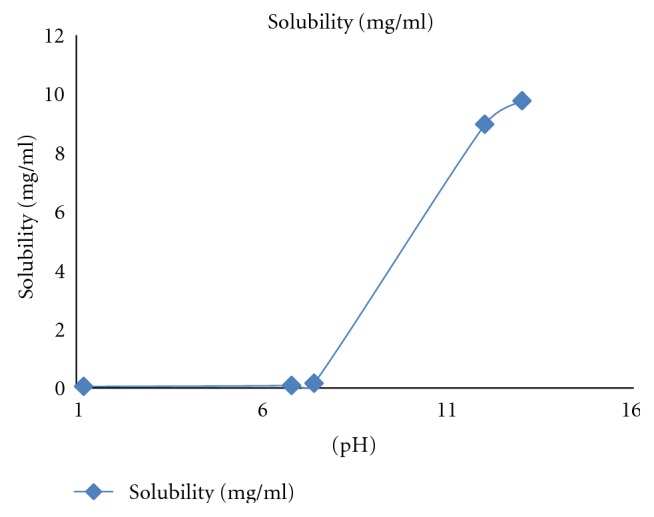
Graphical representation of lornoxicam solubility study at different pH ranges at room temperature.

Solubility of lornoxicam increased substantially with an increase in the pH of media (Figure 1 and Table 2). It was found to be much higher in alkaline pH compared to neutral and acidic one. The solubility was found to be 0.049 mg/mL, 0.082 mg/mL, 0.156 mg/mL, 8.96 mg/mL, and 9.76 mg/mL in media with pH 1.2, pH 6.8, pH 7.4, pH 12, and pH 13, respectively. Lornoxicam showed the maximum solubility in pH 13 as compared to the other pH (Table 2), it is required to find the actual concentration of lornoxicam present in the formulated dual cross-linked beads.

### 4.2. Physical Properties of the Composite Pectin Alginate Beads

Morphological characteristics of pectin alginate beads are demonstrated in Table 3. Average diameter of beads deviated from 1.06 ± 0.07 mm to 1.30 ± 0.03 mm for different batches. Size of formulated beads increases by increasing the content of sodium alginate and cross-linking time. Dual cross-linked beads have more size than single cross-linked beads. Prepared pectin alginate beads are observed to be spherical in shape under the optical microscope. Dual cross-linked beads are found to be more spherical than the single cross-linked beads. It was observed that trivalent ion Al^3+^ has better cross-linking with sodium alginate than divalent ion Ca^2+^.

### 4.3. Fourier Transformed Infrared (FTIR) Study

**Figure 2:**
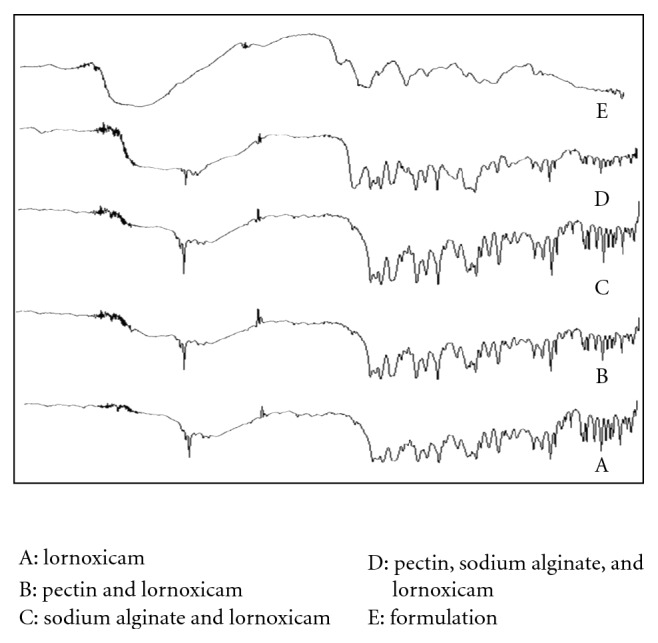
Shows the FTIR spectra of (A) lornoxicam; (B) pectin, and lornoxicam; (C) sodium alginate and lornoxicam; (D) pectin, sodium alginate, and lornoxicam; (E) formulation.

FTIR spectra of pure drug, physical mixture and formulation are reported in Figure 2. FTIR spectra of pure lornoxicam show the peak around 3066 cm^−1^ of –NH group. It shows sharp peak at 1647 cm^−1^ of primary amide and 1598, 1550 of secondary amide. FTIR spectra show at 831 cm^−1^ (aromatic bending), 1147, 1327, 1384 cm^−1^ (−SO_2_ group) and 790 cm^−1^ (C–Cl group). FTIR spectra of pure drug, physical mixture, and formulation are compared. It showed that there is no interaction between the drug and the polymer. So, the drug and polymer are compatible.

### 4.4. Differential Scanning Colorimetry (DSC)

**Figure 3:**
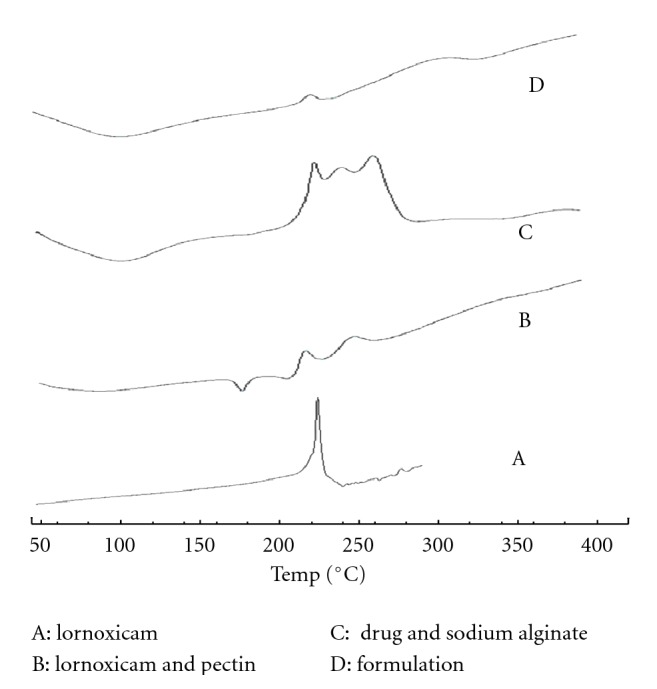
DSC thermograms of (A) lornoxicam (B) lornoxicam, and pectin (C) drug and sodium alginate (D) formulation.

DSC analysis of pure drug, physical mixture and formulation are showed in Figure 3. DSC themogram of lornoxicam showed sharp exothermic peak at 232°C due to its melting process (Figure 3(a)). Drug melting peak is present even in its physical mixture with pectin and sodium alginate. However, the small lowering of peak temperature of related enthalpy variation was observed due to the mixture of component. Physical mixture of lornoxicam and pectin, lornoxicam, and sodium alginate showed the peak temperature at 227°C and 234°C (Figures 3(b) and 3(c)). Pectin alginate beads show peak at 235°C (Figure 3(d)). It indicates that no interaction found between drug and polymers. 

### 4.5. Production Yield and Entrapment Efficiency

Dual cross-linked pectin alginate beads formulation batches P6 and P7 have high drug entrapment efficiency and production yield than the single cross-linking beads formulation P1, P2, P3, P4, P5 have cross-linking time 10 min. But P1 and P4 formulation have high entrapment efficiency amongst these five formulations due to concentration of sodium alginate, suggesting when the concentration of sodium alginate increases, the entrapment efficiency also increases due to more cross-linking between the sodium alginate and cross-linking agents. It indicates that trivalent ion Al^3+^ has greater reactivity than divalent ion Ca^2+^ with carboxyl group of L-guluronic acid units of sodium alginate and pectin. They form more rigid gel matrix structure of beads. Therefore the drug loss gets reduced resulting in better entrapment efficiency.

### 4.6. Effect of Cross-Linking Time

**Figure 4:**
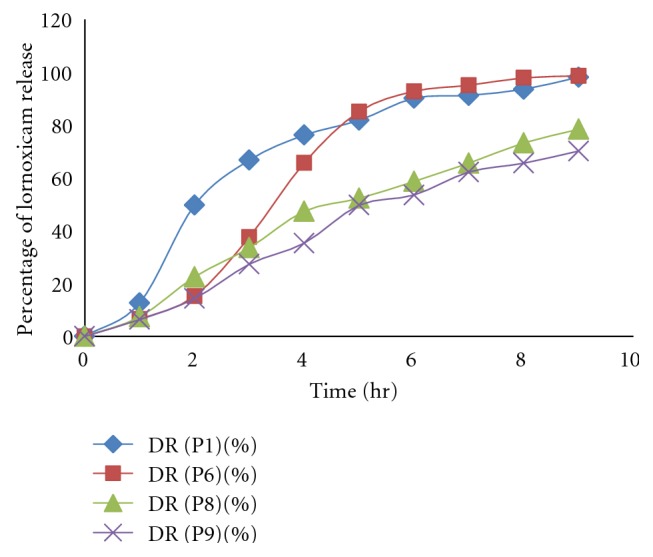
Effect of cross-linking time on release of lornoxicam from pectin alginate beads.

Entrapment efficiency and production yield increase by increasing the cross-linking time (Figure 4). Longer the cross-linking time 10–20 min amount of lornoxicam released from pectin-alginate, beads were reduced and dual cross-linked beads which have cross-linking time 10 min show more sustained release of drug from the pectin alginate beads than single cross-linked beads which have 10 min cross-linking time. Formulation of dual cross-linked beads overcomes the burst release from the formulation. The average size of dual cross-linked beads is found to be more than single cross-linked beads formulation. It suggests that trivalent ion has better cross-linking than divalent ion. 

### 4.7. Swelling Study

**Figure 5:**
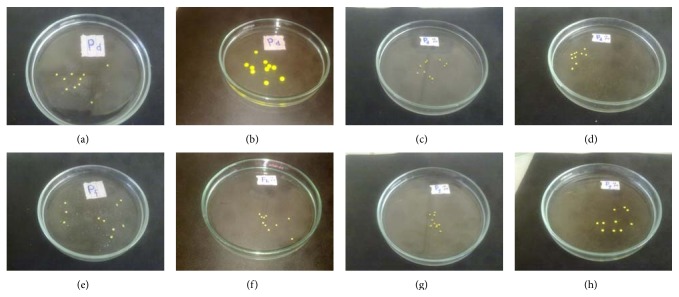
% swelling of single ((a) and (b) in 6.8 pH and (c) and (d) in 1.2 pH) and dual ((e) and (f) in 6.8 pH and (g) and (h) in 1.2 pH) cross-linked beads before ((a), (c), (e), and (g)) and after 2 hours ((b), (d), (f), and (h)).

Swelling property was mostly affected by the concentration of sodium alginate, pectin, and cross-linking of beads. As the concentration of sodium alginate increases the swelling capacity of beads increases considerably while the swelling capacity is rendered by cross-linking (Figure 5). Formulation P1, P4 and P6, P7 have same concentration of sodium alginate but P1 and P4 show higher swelling ability compared to P6 and P7 due to the cross-linking. It is observed that the dual cross-linking beads have lower degree of swelling ability than single cross-linked beads. Also, as the concentration of sodium alginate 450 mg in formulation P4 reduces to 250 mg in P5, the percentage swelling get reduced from 293.8 ± 20.5 to 114.4 ± 22.6 (Table 3). Swelling ability of beads was observed lesser in pH 1.2 compared to pH 6.8.

### 4.8. Scanning Electron Microscopy

**Figure 6:**
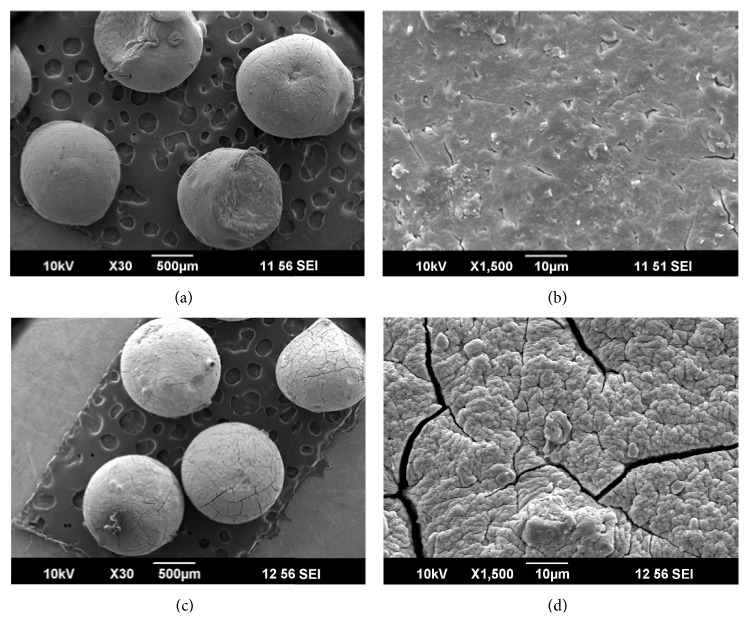
SEM images of single ((a) and (b)) and dual ((c) and (d)) cross-linked beads at different magnifications.

SEM images of the formulated beads at different magnifications were shown in Figure 6. Surface morphology of pectin alginate beads, studied by SEM, indicated that the dual cross-linked beads have more rigid surface with cracks compared to the single cross-linked beads. The dual cross-linked beads are more spherical in shape compared to the single cross-linked beads as shown in SEM image.

### 4.9. *In Vitro* Dissolution Studies

**Figure 7:**
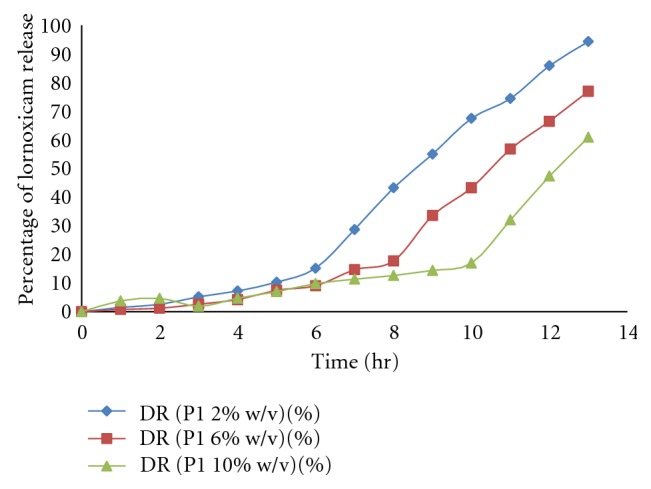
Effect of Eudragit solution concentration on lag time.

USP dissolution apparatus 1 (Basket type) was used to perform the drug release study at 50 rpm and temperature maintained at  37 ± 0.5°C. *In vitro* drug release studies of formulation fabricated with enteric coated as well as uncoated gelatin capsule shell were performed. The bare pectin alginate beads showed immediate drug release while, when the beads are filled in the enteric gelatin capsule there was a pH dependent drug release after the dissolution of the capsule shell. Drug release was inversely related to the concentration of sodium alginate and cross-linking of beads. The decrease in drug release resulted in increase in sodium alginate concentration and increasing the cross-linking time. With the increase in the concentration of the Eudragit S100 and Eudragit L100 in the coating solution there is considerable increase in the lag time for drug release, namely, when 2% Eudragit was used for coating. The lag time was observed to be 6 hrs while 4% and 6% Eudragit showed lag time 8 hrs to 9 hrs, respectively. Hence, we optimized the Eudragit concentration as 2% to get the desired lag time for the drug release (Figure 7). The effective sustained release was obtained from different formulations. The uncoated formulations P1, P4, P6, and P7 showed much sustained drug release than formulation P2, P3, and P5. Moreover, amongst this formulation P6 and P7 gave more sustained drug release due to the formation of the rigid gel structure dual with AlCl^3+^ which reduces the drug release from gel matrix of beads, so overcome the burst release of drug from the pectin alginate beads by formulating the dual cross-linked beads. 

**Figure 8:**
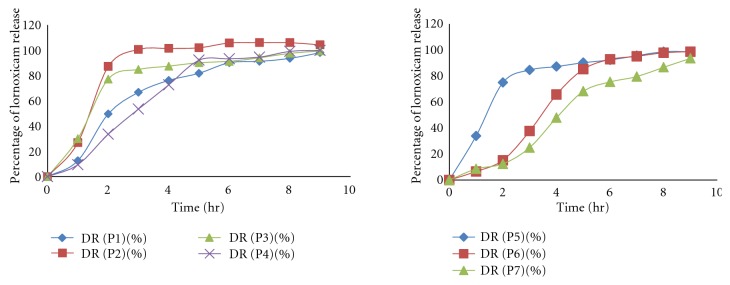
Release profile of lornoxicam from uncoated pectin alginate beads.

**Figure 9:**
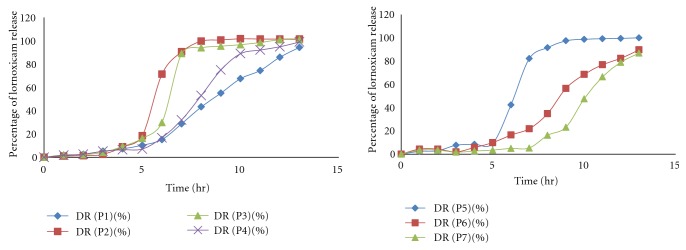
Release profile of lornoxicam from coated pectin alginate beads.

The uncoated formulation P2, P3, and P5 gave burst release in 2 hrs 87.11%, 77.16%, and 74.87% after the swelling of beads (Figure 8). In the case of enteric-coated formulation P1 and P4 formulation depicted 6 hr lag time and P3, P4, and P5 formulation show lag time 5 hr because of lesser concentration of sodium alginate but the formulation P6 and P7 demonstrated 7 hrs to 8 hrs lag time due to dual cross-linking with AlCl^3+^ (Figure 9). In case of dual cross-linking rigid gel structure is formed which reduce the diffusion of drug from beads and increase the lag time of formulation.

## 5. Conclusion

Dual cross-linked pectin alginate beads obtained by ionotropic gelation method were evaluated in the *in vitro* dissolution experiment. The *in vitro* drug release from pectin alginate beads depends on the concentration of sodium alginate, cross-linking ion, and cross-linking time. In case of dual cross-linking drug release, after 7 hrs to 8 hrs lag time which administered at bed time may release lornoxicam when needed most for chronotherapeutics of early morning rheumatoid arthritis attacks in chronic patients. Dual cross-linked beads can be proposed as a promising approach for the design of pulsatile drug delivery system for rheumatoid arthritis.

## Figures and Tables

**Table 1:** Composition of single and dual cross-linked beads formulation.

Batch code	Dispersion phase				Dispersion medium		
	Lornoxicam (mg)	Sodium alginate (mg)	Pectin (mg)	Distilled water (mL)	CaCl_2_ (% w/v)	AlCl_3_ (% w/v)	Cross linking time (min.)
P1	50	400	100	10	5	—	10
P2	50	350	150	10	5	—	10
P3	50	300	200	10	5	—	10
P4	50	450	50	10	5	—	10
P5	50	250	250	10	5	—	10
P6	50	400	100	10	5	5	10
P7	50	450	50	10	5	5	10
P8	50	400	100	10	5	—	15
P9	50	400	100	10	5	—	20

**Table 2:** Solubility of lornoxicam at different pH conditions.

Sr. no.	pH	Wavelength (nm)	Solubility (mg/mL)
1	1.2	365.0	0.049
2	6.8	378.0	0.082
3	7.4	377.0	0.156
4	12	376.5	8.96
5	13	377.5	9.76

**Table 3:** Characteristics of composite pectin alginate beads.

Batch code	Production yield (%)	Entrapment efficiency	Average diameter (mm)	% Swelling (6.8 pH)	% Swelling (1.2 pH)
P1	85.30 ± 0.70	82.95 ±1.42	1.15 ± 0.06	200.5 ± 32.4	31.5 ± 12.3
P2	91.38 ± 1.26	72.93 ± 1.20	1.08 ± 0.05	175.3 ± 27.0	40.8 ± 9.70
P3	87.69 ± 2.39	74.89 ± 1.32	1.06 ± 0.07	158.9 ± 19.5	42.3 ± 13.0
P4	91.49 ± 1.63	79.37 ± 1.55	1.11 ± 0.06	293.8 ± 20.5	24.2 ± 9.13
P5	88.41 ± 0.57	76.69 ± 3.57	1.07 ± 0.05	114.4 ± 22.6	55.2 ± 12.6
P6	93.30 ± 0.76	83.24 ± 1.05	1.30 ± 0.03	57.0 ± 5.1	29.4 ± 9.21
P7	92.70 ± 1.46	83.04 ± 1.57	1.20 ± 0.03	72.6 ± 9.4	25.5 ± 5.49
P8	88.36 ± 1.37	80.65 ± 1.95	1.16 ± 0.04	98.6 ± 11.25	25.6 ± 3.55
P9	90.69 ± 0.98	83.01 ± 1.36	1.18 ± 0.07	81.54 ± 6.28	28.7 ± 5.11
